# S-ketamine plus dexmedetomidine vs. S-ketamine plus propofol for sedation–analgesia during positioning for spinal anesthesia in older adults undergoing lower extremity fracture surgery

**DOI:** 10.3389/fneur.2026.1706118

**Published:** 2026-03-09

**Authors:** Yingying Guo, Tianjiao Wang, Wenyong Han

**Affiliations:** Department of Anesthesiology, Beiiing Electric Power Hospital of State Grid Corporation of China, Beijing, China

**Keywords:** bispectral index, dexmedetomidine, geriatric anesthesia, lower-extremity fracture, positioning, propofol, S-ketamine, spinal anesthesia

## Abstract

**Background:**

Positioning for neuraxial anesthesia in geriatric lower extremity fracture surgery is painful and can destabilize hemodynamics. Sedation–analgesia must balance effective analgesia with respiratory safety and physiologic stability.

**Methods:**

This single-center retrospective cohort study included patients aged ≥65 years undergoing lower extremity fracture surgery under spinal anesthesia who received S-ketamine plus dexmedetomidine (Group A, *n* = 48) or S-ketamine plus propofol (Group B, *n* = 46). Primary outcomes included a numerical rating scale (NRS) positioning pain score of 0–10 and an ordinal posture quality score measured at five stages (T1–T5). Repeated measures were analyzed using mixed-effects models (group, stage, group×stage; random: patient) with covariate adjustment. Stage-wise contrasts were Holm-corrected.

**Results:**

A total of 100 records were screened, and 94 patients were analyzed. Group×stage interactions were significant for pain and posture quality. Group A had lower pain during the most noxious stages (T3–T5; adjusted differences −0.42 to −0.50) and higher odds of better posture quality (OR 2.30–2.70). Physiological differences were modest and stage-specific, with slightly higher SpO_2_ and lower heart rate (HR)/mean arterial pressure (MAP) at T2–T3. Postoperative NRS differed only at 12 h. Adverse events were infrequent; delirium was identified from routine documentation without a standardized instrument (0/48 vs. 2/46).

**Conclusion:**

In this retrospective cohort, dexmedetomidine-based sedation with S-ketamine was associated with improved comfort and cooperation during spinal positioning compared with propofol-based sedation, while adverse events were infrequent in both groups.

## 1 Introduction

Lower extremity fractures are a prevalent and serious concern among older adults, with their incidence expected to rise due to the aging population. These fractures are typically treated surgically to optimize recovery and maintain independence (1, 2). Lower extremity fractures often cause severe pain that complicates perioperative management. Pain at rest is frequently reported as mild to moderate, while pain with movement is often moderate to severe (3). This pain profile underscores the challenges in managing postoperative recovery and highlights the need for effective anesthetic strategies. Neuraxial anesthesia can lead to lower in-hospital mortality and reduced postoperative complications such as pulmonary embolism and infections (4–6). However, the effectiveness of neuraxial anesthesia is contingent upon the patient’s ability to tolerate the necessary positioning, which can be painful for those with hip fractures (7). The choice of anesthesia remains a critical decision in optimizing surgical outcomes and ensuring patient safety, necessitating further research to refine protocols tailored to the needs of the elderly population (8).

Painful positioning in frail elderly patients can trigger a sympathetic activation characterized by a catecholamine surge, leading to increased heart rate (HR) and blood pressure (BP) (9, 10). This stress response pathway can exacerbate cardiac and pulmonary risks, particularly in geriatric patients who are already vulnerable to adverse outcomes from perioperative hemodynamic fluctuations (10, 11). The clinical need for a sedation–analgesia regimen that effectively controls pain while stabilizing hemodynamics during positioning is critical. S-ketamine, dexmedetomidine, and propofol offer distinct pharmacological profiles that can be leveraged for this purpose. S-ketamine, through NMDA antagonism, provides potent analgesia with minimal respiratory depression and a sympathomimetic effect that can support BP (12). Dexmedetomidine, a highly selective α_2_ agonist, offers cooperative sedation and modest analgesia with minimal respiratory depression and sympatholytic effects; any potential neurocognitive benefits remain unclear and were not directly assessed in the present retrospective analysis (12). Propofol, known for rapid and titratable sedation, may cause hypotension and respiratory depression at higher doses, but its combination with S-ketamine can reduce the required dose of propofol, potentially minimizing these side effects (12). A combination of S-ketamine and dexmedetomidine may offer complementary effects by balancing sympathetic tone, providing analgesia, and ensuring cooperative sedation (12). This approach could mitigate the risks associated with hemodynamic instability in elderly patients, thereby improving surgical outcomes and patient safety during procedures that require specific positioning (13, 14).

Comparative evidence remains limited for these two commonly used combinations during the painful positioning phase of neuraxial anesthesia in elderly patients with lower extremity fractures. Accordingly, we compared S-ketamine plus dexmedetomidine with S-ketamine plus propofol during anesthetic positioning, with prespecified endpoints encompassing analgesic efficacy, postural quality, intra-position hemodynamic stability, and safety. We hypothesized that the dexmedetomidine combination would be superior to the propofol combination in achieving better pain control and positioning quality while maintaining greater hemodynamic stability.

## 2 Methods

### 2.1 Study design and ethics

We conducted a single-center, retrospective observational cohort study of older adults undergoing lower extremity fracture surgery under neuraxial anesthesia at Beijing Electric Power Hospital. The protocol conformed to the Declaration of Helsinki and was approved by the Ethics Committee of Beijing Electric Power Hospital (Approval No. 2023083010102).

### 2.2 Setting and participants

The study included consecutive patients aged ≥65 years (including 65) with American Society of Anesthesiologists (ASA) physical status I–III and a body mass index (BMI) of 19.5–26.2 kg/m^2^ who underwent elective lower extremity fracture surgery with planned spinal anesthesia during the study period at our institution. Patients were excluded if they (1) had a documented contraindication to propofol, esketamine (S-ketamine), or dexmedetomidine; (2) lacked key peri-positioning data; (3) had psychiatric disorders that prevented reliable assessment; (4) were simultaneously enrolled in interventional studies that could influence sedative choice; or (5) if intraoperative blood loss exceeded 400 mL or the procedure lasted longer than 4 h. The final analytic cohort comprised 94 patients after screening the hospital information system and anesthesia records.

### 2.3 Exposure definition and clinical protocols

Exposure was the sedative–analgesic regimen used during positioning for neuraxial anesthesia as delivered in routine care. Assignment to a regimen reflected the attending anesthesiologist’s preference and drug availability within departmental practice. According to institutional protocol, all patients received a single intravenous bolus of S-ketamine 0.5 mg/kg approximately 10 min before positioning. Subsequently, one of two infusion strategies was used and titrated in real time to maintain a BIS between 60 and 80 together with stable hemodynamics: dexmedetomidine at 0.2–0.6 μg/kg/h (Group A) or propofol at 0.5–4 mg/kg/h (Group B). Infusion rates were adjusted at the discretion of the anesthesiologist according to BIS, blood pressure, and heart rate; standard fasting (8 h solids, 4 h clear liquids) and monitoring were applied, including continuous ECG, heart rate, non-invasive blood pressure, peripheral oxygen saturation, and radial arterial catheterization for invasive pressure measurement, and study infusions were discontinued upon skin closure. Regimen selection reflected the attending anesthesiologist’s preference and drug availability within departmental practice. Primary outcomes were assessed during positioning for neuraxial placement before surgical incision, and the surgical team was not involved in sedation selection or delivery during the positioning/spinal placement period.

Postoperative delirium was identified from routine clinical documentation (physician diagnosis and/or nursing chart notes indicating delirium/acute confusion) without the use of a standardized delirium instrument.

### 2.4 Neuraxial anesthesia and positioning

After entry to the operating room and initiation of monitoring, patients were positioned with the fractured limb uppermost in the lateral decubitus position and then guided into hip and knee flexion to facilitate spinal placement. Spinal anesthesia was performed at the L2–3 or L3–4 interspace using a standard technique; upon free flow of cerebrospinal fluid, isobaric ropivacaine 0.33% (10–15 mg) was injected intrathecally, and the sensory block was managed below the T10 dermatome throughout the surgery. The sequence of peri-positioning assessments followed five predefined time points corresponding to room entry (T1), lateral decubitus (T2), hip/knee flexion (T3), dural puncture (T4), and intrathecal drug administration (T5).

### 2.5 Postoperative analgesia and PACU care

All patients received a standardized postoperative patient-controlled analgesia regimen consisting of sufentanil 50 μg and flurbiprofen axetil 200 mg diluted to 100 mL with normal saline, delivered with a background rate of 2 mL/h, a 15-min lockout, and a 2-mL bolus. In the post-anesthesia care unit, hypotension was defined as systolic blood pressure <90 mmHg, with ephedrine 3–5 mg administered if systolic pressure fell <80 mmHg or declined >30% from baseline. Oxygen desaturation was defined as SpO_2_ < 90% and treated by increasing face mask oxygen flow to 6 L/min; rescue analgesia for NRS > 4/10 consisted of sufentanil 5 μg intravenously, repeatable once after 10 min if required.

### 2.6 Outcomes and variable definitions

The co-primary outcomes were positioning pain evaluated with the 0–10 NRS and postural quality during positioning assessed with an institutional ordinal posture quality score (range 1–4; higher values indicate better positioning/cooperation; Supplementary Table S1). Secondary outcomes were physiologic responses and perioperative logistics, including HR, MAP, SpO_2_, and BIS recorded at T1–T5; anesthesia duration; number of lumbar puncture attempts; the interval from lateral positioning to intrathecal injection (anesthesia positioning time); surgery duration; postoperative pain measured by the 0–10 numerical rating scale (NRS) at 2 h, 4 h, 12 h, and 24 h; patient-controlled analgesia usage and residual volume; and adverse events, including hypotension, oxygen desaturation, and postoperative delirium as documented by the clinical team without a standardized delirium instrument.

### 2.7 Data sources and collection

Data were obtained from the electronic medical record, the anesthesia information management system, BIS monitor logs, and standardized nursing and anesthetic charts. For each case, demographic characteristics, comorbidities, ASA class, BMI, intraoperative hemodynamics, sedative dosing and changes, NRS and posture quality assessments at T1–T5, and postoperative outcomes were transcribed into an analysis dataset using a predefined data dictionary.

### 2.8 Statistical analysis

Continuous variables were screened for distributional assumptions using histograms, Q–Q plots, and Shapiro–Wilk tests and are summarized as mean ± SD when approximately normal and as median (IQR) otherwise. Categorical variables are summarized as n (%). Baseline comparability was assessed descriptively with standardized mean differences (SMDs) rather than hypothesis tests. We considered |SMD| < 0.10 as negligible imbalance. No baseline *p*-values were used for inference. Analyses were performed using the R package (version 4.4).

Since outcomes were measured repeatedly across five intra-procedural stages (T1–T5), we used mixed-effects models that account for within-patient correlation. For continuous outcomes (NRS positioning pain, heart rate, mean arterial pressure, SpO_2_, and BIS), we used linear mixed-effects models with a random intercept for patient and fixed effects for group, stage, and group×stage interaction. For the ordinal posture quality score, we used a cumulative logit mixed model (proportional odds link) with the same fixed and random effects. All models were adjusted for prespecified covariates [age, sex, BMI, ASA class, and major comorbidities: coronary artery disease, chronic heart failure, hypertension, atrial fibrillation, chronic obstructive pulmonary disease (COPD), and obstructive sleep apnea (OSA)]. We also adjusted for the baseline value of the outcome at the participant level (ANCOVA style). Model parameters were estimated by restricted maximum likelihood (linear models) and maximum likelihood (ordinal models). The primary test for each repeated-measures endpoint was the group×stage interaction (two-sided *α* = 0.05). When the interaction was significant, we estimated stage-specific between-group contrasts at each stage and controlled the family-wise error within that endpoint across the five stages using the Holm procedure. For continuous outcomes, effects are reported as adjusted mean differences with 95% CIs derived from the model-based estimated marginal means (EMMs); for posture, effects are adjusted odds ratios with 95%.

For outcomes recorded once per patient procedural metrics (time to optimal positioning, surgery duration, and distribution of lumbar puncture attempts), postoperative NRS at 2 h, 4 h, 12 h, and 24 h, and adverse events, we summarized between-group differences as mean differences with 95% CIs and Welch’s two-sample t-tests for continuous variables and as risk ratios with 95% CIs and Fisher’s exact tests for binary variables. When a cell count was zero, we applied the Haldane–Anscombe correction (adding 0.5 to each cell) and computed CIs on the log scale. For the distribution of one, two, and three puncture attempts, we used a Pearson χ^2^ test across categories. Since drug assignment was protocol-defined, no formal hypothesis tests were performed for agents used exclusively in one arm (dexmedetomidine and esketamine in Group A; propofol in Group B); propofol dose comparisons were presented descriptively to reflect exposure differences by design.

## 3 Results

**Figure 1:**
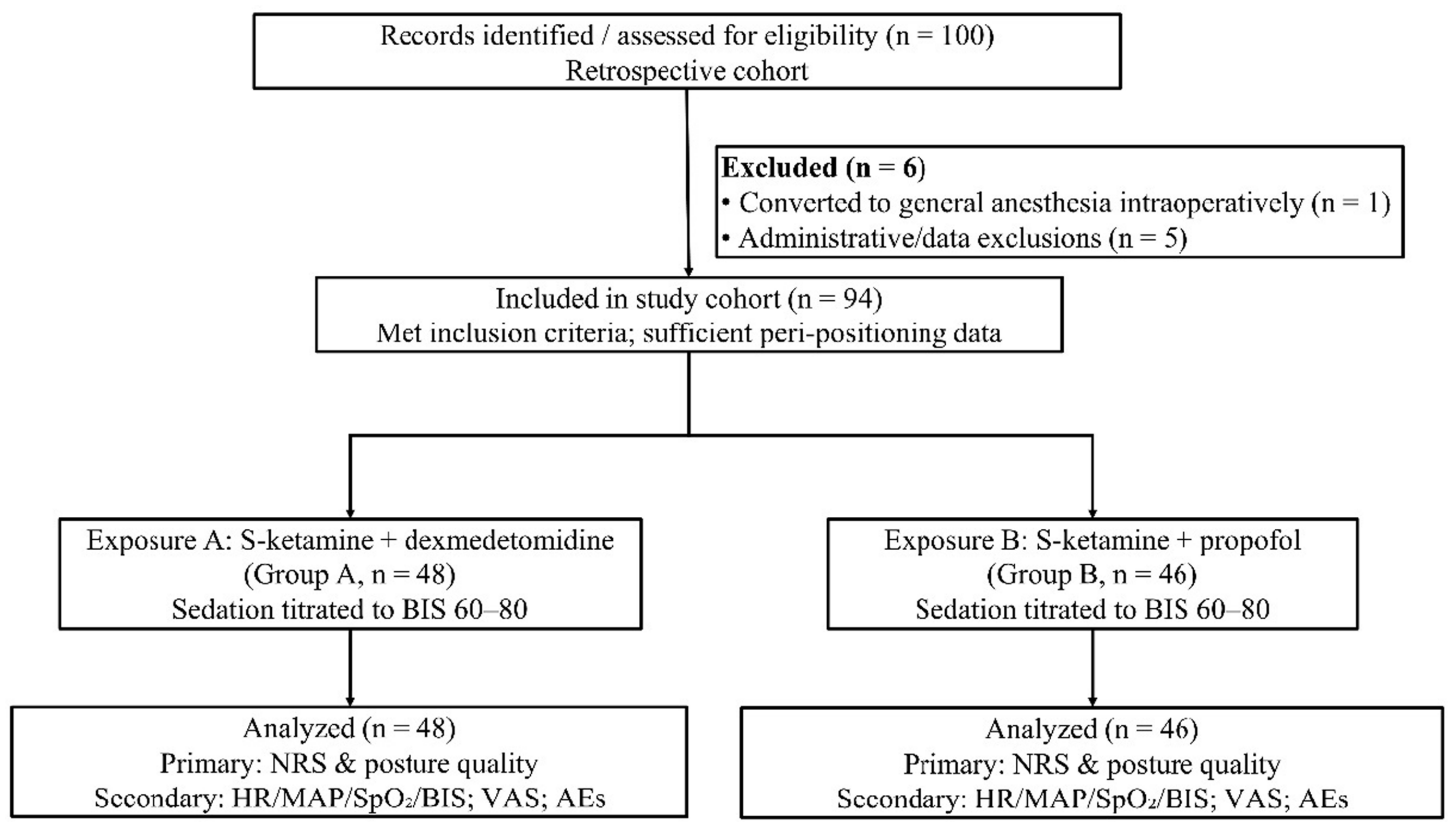
Study cohort flow diagram. A total of 100 records were assessed with 6 of them excluded (1 conversion to general anesthesia, 5 administrative/data exclusions). 94 patients were included and classified by exposure—*S*-ketamine plus dexmedetomidine (Group A, *n* = 48) and *S*-ketamine plus propofol (Group B, *n* = 46)—with all included in the analysis.

A total of 100 records were screened, of which 6 were excluded (3 for not meeting the inclusion criteria, 2 for missing key intra-stage measurements, and 1 for conversion to general anesthesia before positioning). The remaining 94 patients were included in the study, and all were analyzed, with 48 patients in Group A (dexmedetomidine ± esketamine) and 46 patients in Group B (propofol; Figure 1).

Baseline demographics, comorbidities, and pre-positioning physiologic measures were similar between the groups (Table 1). Standardized mean differences were <0.10 for all variables, including baseline pain (NRS 3.00 vs. 3.01), heart rate (78.2 vs. 78.6 beats/min), mean arterial pressure (90.1 vs. 90.3 mmHg), oxygen saturation (98.4% vs. 98.3%), and BIS (85.0 vs. 84.9), indicating no meaningful imbalance before adjustment.

**Table 1:** Baseline characteristics.

Characteristic	Group A (*n* = 48)	Group B (*n* = 46)	SMD
Age (years), mean ± SD	76.2 ± 6.8	76.1 ± 7.0	0.01
Female, n/N (%)	27/48 (56.3%)	25/46 (54.3%)	0.04
BMI (kg/m^2^),mean ± SD	23.2 ± 2.1	23.3 ± 2.0	0.05
ASA class, n			—
II	11	10	
III	33	32	
IV	4	4	
Hypertension, n/N (%)	29/48 (60.4%)	27/46 (58.7%)	0.04
CAD, n/N (%)	12/48 (25.0%)	12/46 (26.1%)	0.03
Diabetes, n/N (%)	10/48 (20.8%)	9/46 (19.6%)	0.03
COPD/OSA, n/N (%)	7/48 (14.6%)	7/46 (15.2%)	0.02
Atrial fibrillation, n/N (%)	5/48 (10.4%)	5/46 (10.9%)	0.02
Baseline NRS (0–10), mean ± SD	3.00 ± 1.09	3.01 ± 1.07	0.01
Baseline HR (beats/min), mean ± SD	78.2 ± 10.3	78.6 ± 9.8	0.04
Baseline MAP (mmHg), mean ± SD	90.1 ± 11.5	90.3 ± 11.2	0.02
Baseline SpO₂ (%), mean ± SD	98.4 ± 0.9	98.3 ± 1.0	0.10
Baseline BIS, mean ± SD	85.0 ± 5.8	84.9 ± 5.9	0.02

SMD, standardized mean difference. No clinically meaningful imbalances (all |SMD| < 0.10).

Consistent with the statistical plan, repeated measures were analyzed using mixed-effects models including fixed effects for group, stage (T1–T5, categorical), and group×stage interaction, with a patient-level random intercept, covariate adjustment, and Holm correction across stages. For positioning pain, the between-group separation was stage-specific: differences were minimal at T1–T2 and emerged during hip/knee flexion and spinal placement (T3–T5) with adjusted mean differences (A–B) of −0.42 at T3 (95% CI − 0.59 to −0.25), −0.50 at T4 (−0.67 to −0.33), and −0.48 at T5 (−0.65 to −0.30; Table 2A). For posture quality, a similar pattern was observed: odds of achieving a higher posture grade favored Group A at T3–T5 (adjusted ORs 2.30–2.70), while T1–T2 showed minimal differences (Table 2B).

**Table 2:** Primary outcomes.

Stage	Difference or OR	95% CI	Holm-adj p
A. NRS positioning pain (0–10)	Difference		
T1	0.00	−0.15 to 0.15	0.98
T2	−0.08	−0.24 to 0.07	0.29
T3	−0.42	−0.59 to −0.25	<0.001
T4	−0.50	−0.67 to −0.33	<0.001
T5	−0.48	−0.65 to −0.30	<0.001
B. Posture quality	OR	95% CI	Holm-adj p
T1	1.08	0.68 to 1.73	0.78
T2	1.20	0.77 to 1.87	0.56
T3	2.30	1.36 to 3.90	0.006
T4	2.70	1.57 to 4.63	0.003
T5	2.60	1.47 to 4.58	0.004

Models adjust for age, sex, BMI, ASA, prespecified comorbidities, and T1 baseline; random intercept for patient; Holm control across T1–T5 within outcomes.

Secondary physiologic response showed modest, stage-specific differences concentrated during early positioning (T2–T3), while other stages were similar (Table 3). Compared with Group B, Group A had slightly lower HR (approximately 2–4 beats/min lower at T2–T3) and slightly lower MAP (approximately 3 mmHg lower at T2–T3). Group A also had slightly higher SpO_2_ at T2–T4 (differences <1 percentage point) and slightly lower BIS at T2–T3 (differences ~2–4 units). Full model-based estimates with 95% CIs and Holm-adjusted *p*-values are reported in Table 3.

**Table 3:** Secondary intra-procedural physiology.

Stage	Difference	95% CI	Holm-adj p
A. Heart rate (beats/min)			
T1	−0.4	−2.3 to 1.5	0.82
T2	−4.0	−6.1 to −1.9	0.006
T3	−2.5	−4.8 to −0.3	0.042
T4	−1.1	−3.1 to 0.9	0.41
T5	−0.6	−2.5 to 1.3	0.71
B. Mean arterial pressure (mmHg)			
T1	−0.7	−2.6 to 1.2	0.62
T2	−3.2	−5.3 to −1.1	0.007
T3	−2.8	−4.9 to −0.7	0.009
T4	−1.4	−3.4 to 0.6	0.24
T5	−0.5	−2.2 to 1.2	0.74
C. Oxygen saturation (%)			
T1	+0.1	−0.3 to +0.5	0.65
T2	+0.7	+0.3 to +1.1	0.006
T3	+0.8	+0.4 to +1.2	0.004
T4	+0.6	+0.2 to +1.0	0.008
T5	+0.1	−0.3 to +0.5	0.74
D. Bispectral index			
T1	−0.6	−2.7 to +1.4	0.68
T2	−3.8	−5.9 to −1.7	0.004
T3	−2.5	−4.9 to −0.1	0.038
T4	−1.3	−3.4 to +0.8	0.31
T5	−0.7	−2.6 to +1.2	0.72

Procedure-level metrics were similar between the groups (Table 4), including time to optimal positioning, surgery duration, and the distribution of lumbar puncture attempts.

**Table 4:** Procedural efficiency and technical metrics.

Metric	Group A (mean ± SD)	Group B (mean ± SD)	Difference (A–B)	95% CI	*p*-value
Time to optimal positioning, min	4.2 ± 1.2	4.3 ± 1.2	−0.10	−0.59 to +0.39	0.686
Surgery duration, min	80.5 ± 19.9	82.4 ± 19.5	−1.90	−9.87 to +6.07	0.640
Lumbar puncture attempts, n			—	—	0.934^†^
1	34	31			
2	12	13			
3	2	2			

† Pearson χ^2^ across one, two, and three attempts (two-sided).

Postoperative NRS values were similar at 2 h and 4 h. A small between-group difference was observed at 12 h, while values converged again by 24 h (Table 5).

**Table 5:** Postoperative pain (NRS, 0–10).

Time after surgery	Group A (mean ± SD)	Group B (mean ± SD)	Difference (A–B)	95% CI	*p*-value
2 h	2.0 ± 1.0	2.2 ± 1.1	−0.20	−0.63 to +0.23	0.357
4 h	1.8 ± 1.0	2.0 ± 1.1	−0.20	−0.63 to +0.23	0.357
12 h	1.1 ± 0.8	1.7 ± 1.0	−0.60	−0.97 to −0.23	0.001
24 h	1.0 ± 0.8	1.1 ± 0.9	−0.10	−0.44 to +0.24	0.570

As expected from the assigned regimens, Group A received dexmedetomidine and esketamine, and Group B received propofol with no crossover. Propofol exposure was substantially lower in Group A (Table 6).

**Table 6:** Sedation or adjunct medication use.

Variable	Group A (mean ± SD)	Group B (mean ± SD)	Difference (A–B)	95% CI	*p*-value
Propofol, total mg	8 ± 15	150 ± 50	−142	−157.1 to −126.9	<0.001
Dexmedetomidine, total μg	40 ± 10	0	—	—	—
Esketamine, total mg	15 ± 5	0	—	—	—
Dexmedetomidine infusion duration, min	30 ± 10	—	—	—	—
Propofol infusion duration, min	—	30 ± 12	—	—	—

No statistical comparison for drugs given exclusively by design to one group.

Adverse event rates were low and similar between the groups (Table 7). Hypotension occurred in 12.5% vs. 17.4% (risk ratio: 0.72, 95% CI: 0.27–1.91) and desaturation in 2.1% vs. 8.7% (0.24, 0.03–2.06) for Groups A and B, respectively. Postoperative delirium was not observed in 48 patients in Group A, and it was observed in 2 of 46 patients in Group B (risk ratio: 0.19, 95% CI: 0.01–3.89), with no other pattern suggesting differential harm.

**Table 7:** Adverse events.

Event	Group A (n = 48)	Group B (n = 46)	Risk ratio (A/B)	95% CI	*p*-value
Hypotension	6 (12.5%)	8 (17.4%)	0.72	0.27 to 1.91	0.571
Desaturation	1 (2.1%)	4 (8.7%)	0.24	0.03 to 2.06	0.199
Bradycardia	4 (8.3%)	3 (6.5%)	1.28	0.30 to 5.40	1.000
Tachycardia	2 (4.2%)	5 (10.9%)	0.38	0.08 to 1.88	0.263
PONV	3 (6.3%)	4 (8.7%)	0.72	0.17 to 3.04	0.711
Delirium (POD)	0 (0%)	2 (4.3%)	0.19‡	0.01 to 3.89	0.237

‡ Haldane–Anscombe correction applied for RR/CI (zero events in Group A).

## 4 Discussion

In this retrospective cohort of older adults undergoing lower extremity fracture surgery under spinal anesthesia, the S-ketamine plus dexmedetomidine regimen was associated with a stage-dependent improvement in comfort and cooperation during positioning. Differences were minimal at room entry and lateral positioning (T1–T2) but emerged during hip/knee flexion and spinal placement (T3–T5), where pain scores were lower and posture quality ratings were higher. Physiologic differences were small in absolute magnitude and largely concentrated during early positioning (T2–T3), while procedure-level metrics were similar between the groups.

The superiority of S-ketamine plus dexmedetomidine in our cohort likely reflects complementary pharmacodynamics: *α*₂-mediated analgesia, cooperative sedation, and sympatholysis from dexmedetomidine with minimal respiratory depression (15–20), paired with S-ketamine’s anti-hyperalgesia and sympathomimetic pressor that may reduce movement-evoked discomfort during positioning (19, 21). Clinically, between-group differences emerged during the most painful stages (T3–T5), when improved comfort and cooperation are most likely to facilitate spinal placement. Physiologic differences were modest in magnitude and concentrated at early positioning (T2–T3): compared with the propofol regimen, the dexmedetomidine regimen was associated with slightly lower HR and MAP and slightly higher SpO_2_. BIS values were maintained within the targeted range in both groups (20). The observed between-group differences at T2–T3 (approximately 2–4 BIS units) likely reflect small differences in sedation depth and agent-specific EEG effects and should not be interpreted as evidence of neuroprotection. We also noted less total sedative use, lower 12-h NRS, and two delirium cases only in the propofol group, which aligned with dexmedetomidine’s airway-sparing profile in patients with coronary artery disease, chronic heart failure, hypertension, atrial fibrillation, COPD, and OSA (16, 20). In contrast, the vasodilatory and respiratory-depressant effects of propofol can become dose-limiting under painful stimuli—even when combined with S-ketamine—leaving residual risks of hypotension and oxygen desaturation (20, 21).

Compared with general anesthesia, neuraxial anesthesia in older adults has been linked to lower in-hospital mortality and shorter length of stay (22) and to fewer complications such as acute myocardial infarction, hypotension, and cognitive dysfunction (5, 23). However, positioning pain remains a practical barrier to neuraxial techniques—particularly in patients with cardiopulmonary disease—because lateral decubitus and hip/knee flexion can provoke nociceptive surges and hemodynamic swings (24). Adjunctive regional analgesia can help (e.g., PENG block vs. fascia iliaca compartment block) to facilitate positioning and improve early analgesia (6), but systematic evidence specifically isolating the positioning phase has been scarce. A recent study has reported better ease of spinal positioning and lower pain scores with pericapsular nerve group (PENG) block compared with supra-inguinal fascia iliaca block in hip fracture patients undergoing spinal anesthesia. These data reinforce the central clinical premise that optimizing movement-evoked pain improves positioning success, whether via regional techniques or systemic sedation–analgesia (25). At the same time, accumulating data suggest that dexmedetomidine can enhance sleep architecture and reduce postoperative delirium in older adults—including those with depression and insomnia—with randomized and cohort evidence indicating reductions in sleep disturbance and delirium burden (26–30). Finally, while S-ketamine-based combinations can spare hypnotic doses, they may not fully offset propofol-related circulatory effects at higher rates (31). Against this backdrop, our study indicated that the S-ketamine plus dexmedetomidine strategy improves comfort and physiologic stability exactly when neuraxial placement is attempted.

Clinically, for geriatric lower extremity fracture patients—including those with coronary artery disease and COPD—a pre-positioning regimen of S-ketamine 0.5 mg/kg IV followed by dexmedetomidine 0.2–0.6 μg/kg/h, titrated to BIS 60–80, can improve positioning success, patient comfort, and physiologic stability (20, 31, 32). In our study, this approach reduced rescue sedative needs, preserved MAP/HR/SpO_2_ during positioning, and yielded lower 12-h pain scores, all without excess adverse events relative to the comparator. Whether improved comfort during positioning and modest changes in physiologic variables translate into better neurocognitive outcomes remains unclear and should be evaluated prospectively using standardized delirium assessments (26–29). Integration with standard monitoring and multimodal perioperative analgesia is advisable, and the regimen is especially attractive when painful positioning is anticipated or regional blocks are unavailable or insufficient (33). Implementation should include vigilant hemodynamic and respiratory surveillance and non-pharmacologic delirium prevention measures to maximize safety and recovery in this vulnerable population (31).

This study has several limitations that temper inference. First, the retrospective, single-center design with clinician-driven selection of dexmedetomidine or propofol precludes causal conclusions and introduces potential selection and performance biases. Although we used covariate-adjusted mixed-effects models (including group, stage, and group×stage interaction with patient random effects) to address repeated measurements and adjust for measured baseline characteristics, residual confounding from unmeasured factors (e.g., frailty, fracture severity, preoperative analgesic exposure, time-of-day effects, and other care-process variables) may persist. Surgeon identifiers were not consistently available in the extracted dataset; therefore, we could not evaluate surgeon-level clustering or surgeon–risk interactions, and case-mix differences related to surgical practice cannot be excluded. Second, outcome ascertainment relied on routine clinical documentation without blinding. The posture quality measure is an institutional ordinal scale rather than a universally validated instrument, which may limit external comparability and introduce potential measurement variability between providers. In addition, postoperative delirium was identified from chart documentation rather than a standardized delirium instrument. Given the very low event counts, safety outcomes should be considered descriptive/exploratory. The sample size was modest and drawn from a single institution with a relatively narrow distribution of patient characteristics and local practice patterns, limiting generalizability to other settings and to more complex or higher-risk populations. Sedation was titrated in real time to a target BIS range at clinician discretion. We did not model time-varying exposure or dose–response relationships, and BIS itself may be influenced by agent-specific EEG effects. Finally, while Holm correction controlled multiplicity across the five stage-wise contrasts within each repeated-measures endpoint, we did not apply a global multiplicity adjustment across multiple secondary endpoints; therefore, secondary physiologic findings should be interpreted cautiously as supportive patterns rather than definitive confirmatory outcomes.

In summary, a dexmedetomidine-based positioning strategy (with an optional small esketamine bolus) yielded better positioning analgesia and posture quality, with lower HR/MAP and higher SpO_2_ during the most noxious stages and a lower 12-h postoperative pain score compared with a propofol-based strategy without esketamine. These observational findings support considering dexmedetomidine-based positioning sedation as a preferred approach for similar geriatric populations undergoing neuraxial anesthesia and warrant prospective randomized or pragmatic comparative trials—including regimens with regional positioning blocks—to confirm effectiveness and safety.

## Data Availability

The raw data supporting the conclusions of this article will be made available by the authors, without undue reservation.
