# Supplementary material for: S-ketamine plus dexmedetomidine vs. S-ketamine plus propofol for sedation–analgesia during positioning for spinal anesthesia in older adults undergoing lower extremity fracture surgery
Source: Front Neurol. 2026 Mar 9;17:1706118. doi: 10.3389/fneur.2026.1706118 (PMC13006294; doi:10.3389/fneur.2026.1706118)
Supplement: Supplementary file 1 [file Table_1.docx]

**Table S1. Definition of institutional posture-quality score**

| **Score** | **Definition (anchor)** |
| --- | --- |
| 1 | Poor: unable to achieve/maintain required position; repeated interruption due to pain or noncooperation; positioning inadequate for spinal placement without substantial assistance. |
| 2 | Fair: position achieved only with substantial assistance/repeated instructions; frequent adjustments required. |
| 3 | Good: position achieved with minimal assistance; tolerates spinal placement with minor adjustments. |
| 4 | Excellent: ideal positioning achieved smoothly and maintained independently with minimal/no assistance. |
